# T2* assessment of the three coronary artery territories of the left ventricular wall by different monoexponential truncation methods

**DOI:** 10.1007/s10334-022-01008-4

**Published:** 2022-04-18

**Authors:** Pandji Triadyaksa, Jelle Overbosch, Matthijs Oudkerk, Paul Eduard Sijens

**Affiliations:** 1 University of Groningen, 9700 RB Groningen, The Netherlands; 2 Departemen Fisika, Universitas Diponegoro, Fakultas Sains Dan Matematika, Prof. Sudharto street, Semarang, 50275 Indonesia; 3 Department of Radiology, University of Groningen, University Medical Center Groningen, EB45, PO Box 30001, 9700 RB Groningen, The Netherlands; 4 Institute for Diagnostic Accuracy, Groningen, The Netherlands

**Keywords:** Myocardial iron deposition, Bright blood multi-gradient echo, Pixel-wise T2*, R^2^ truncation method, SNR truncation method, Coronary artery territories

## Abstract

**Objectives:**

This study aimed at evaluating left ventricular myocardial pixel-wise T2* using two truncation methods for different iron deposition T2* ranges and comparison of segmental T2* in different coronary artery territories.

**Material and methods:**

Bright blood multi-gradient echo data of 30 patients were quantified by pixel-wise monoexponential T2* fitting with its *R*^2^ and SNR truncation. T2* was analyzed at different iron classifications. At low iron classification, T2* values were also analyzed by coronary artery territories.

**Results:**

The right coronary artery has a significantly higher T2* value than the other coronary artery territories. No significant difference was found in classifying severe iron by the two truncation methods in any myocardial region, whereas in moderate iron, it is only apparent at septal segments. The *R*^2^ truncation produces a significantly higher T2* value than the SNR method when low iron is indicated.

**Conclusion:**

Clear T2* differentiation between the three coronary territories by the two truncation methods is demonstrated. The two truncation methods can be used interchangeably in classifying severe and moderate iron deposition at the recommended septal region. However, in patients with low iron indication, different results by the two truncation methods can mislead the investigation of early iron level progression.

## Introduction

Cardiac magnetic resonance imaging (MRI) by multi-gradient echo (MGE) has become a widespread method for the non-invasive assessment of myocardial iron deposition [1–3]. Myocardial iron deposition assessment is essential for treatment decisions in patients with thalassemia, hemochromatosis, cardiomyopathy, and sickle cell disease [3–6]. The deposition quantification is conducted by applying T2* evaluation on bright blood mode MGE image series [1, 2, 4, 7–13] at mid-ventricular septal [2, 10, 14] or global myocardium [1, 4, 8]. Several post-mortem studies have verified the relation between cardiac iron deposition with T2* value [7, 9, 11, 15, 16]. On its evaluation, iron distribution is found at different circumferential myocardial regions [4, 11, 17]. Therefore, the segmental approach is suggested to be more sensitive in detecting the early progression of iron distribution than the evaluation of the septum only.

**Fig. 1:**
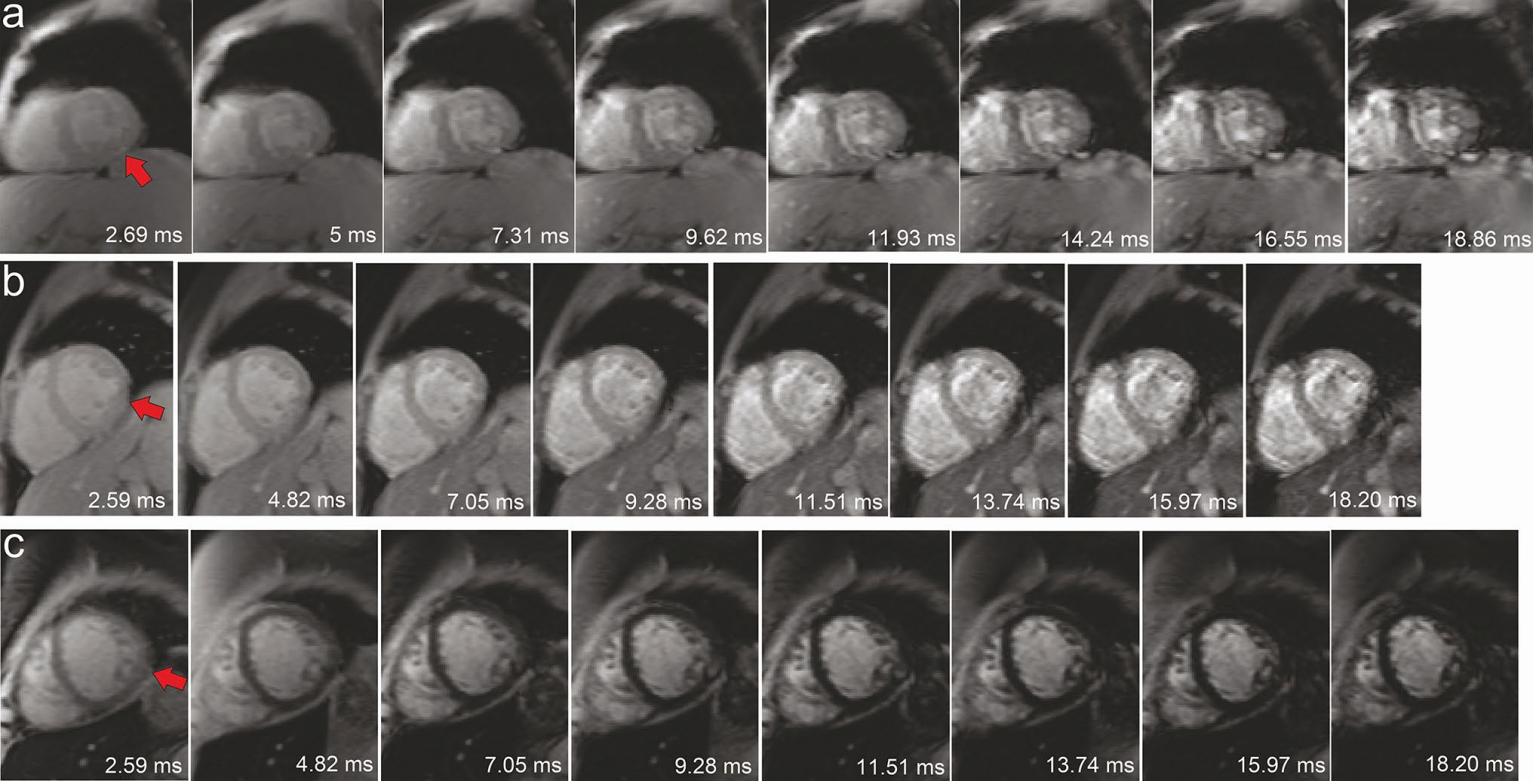
Multi-gradient echo image series at mid-ventricular slice (**a**–**c**) indicates susceptibility artifact at inferolateral (red arrow)

The established myocardial T2* is assessed by either pixel-wise quantification [7, 11, 12, 18, 19] or per region of interest [1, 2, 4, 8–10]. In their methods, pixel-wise quantification has the advantage of identifying the heterogeneity of iron distribution [7, 12] but comes with the drawback of it being prone to susceptibility artifact [13, 19] as located in the MGE image series in Fig. 1. Therefore, in pixel-wise T2* quantification, a study suggests using the median rather than mean T2* values to characterize myocardial segments [12].

In the bright blood mode, signal plateau or offset can occur at longer TEs. This offset is due to noise bias, heterogeneous iron distribution in myocytes, signal offset, myocardial blood volume, motion artifact, or iron-poor tissue contributions [20]. Therefore, one might suggest adding an offset constant C to the classic monoexponential equation [20]. However, in the presence of artifacts, an increase by a constant value will consequently reduce the initial signal intensity estimation of the monoexponential equation and, therefore, lower the T2* estimation. This estimation leads to a biased interpretation of iron deposition, especially at low iron concentrations, as reported in model and patient data [15, 21, 22].

Another way to handle the signal plateau is by truncating higher TE data when the signal intensity reaches the noise level [15, 18], known as signal to noise ratio (SNR) truncation. Evaluating the goodness of the fit (*R*^2^), known as the *R*^2^ truncation method, can also be a criterion to exclude data points at longer TEs [10, 12]. Comparisons between either truncation method with an offset method were conducted previously [15, 22, 23]. Nevertheless, no comparison was conducted between truncation methods raising the question of which method best replaces the classic monoexponential method in quantifying myocardial T2* from clinical MGE data.

**Fig. 2:**
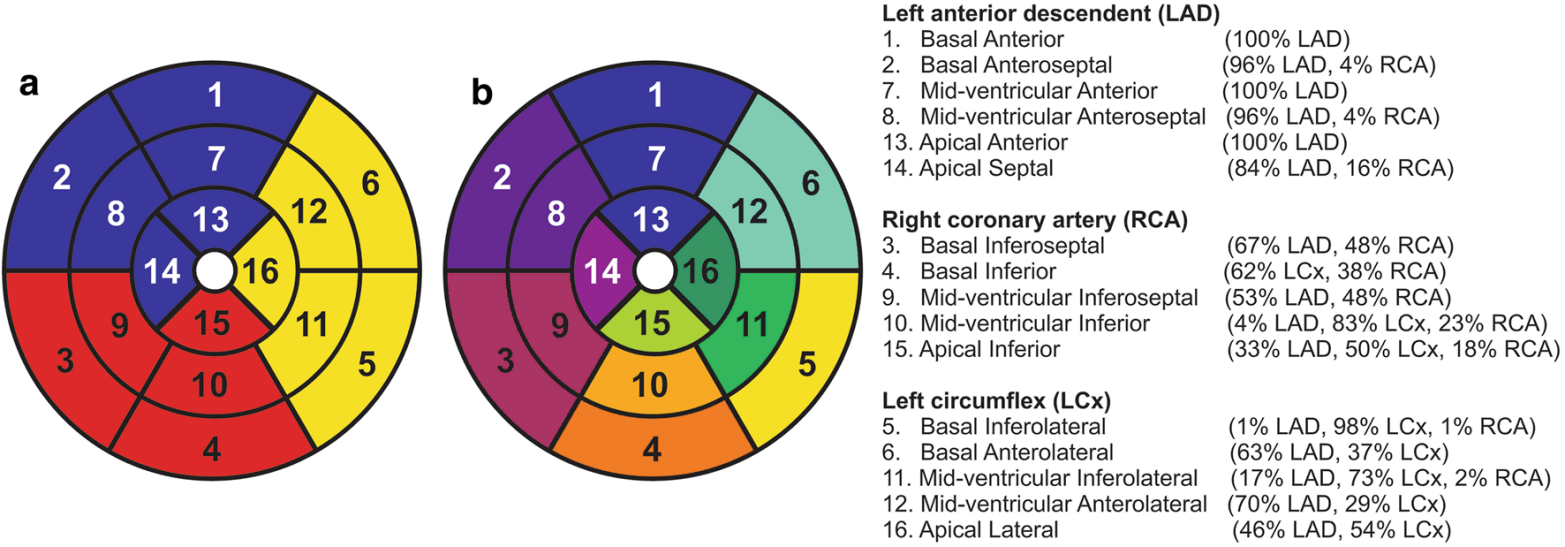
The coronary artery blood supply on the circumferential regions according to the American Heart Association 16-segment model [25] (**a**) and further information of the percentage of the coronary arteries contribution for each segment [26] (**b**)

It is known that different coronary arteries supply different circumferential regions of the left ventricle (LV) [24], as shown in the American Heart Association (AHA) 16-segment model, reproduced in Fig. 2a [25]. Detailed analysis of the artery supply, reproduced in Fig. 2b, shows that most myocardial segments are served by more than one coronary artery [26]. Therefore, this study aimed to compare the SNR and *R*^2^ truncation methods in evaluating the pixel-wise T2* value for LV myocardial iron detection and analyze and compare the T2* values of the different coronary artery territories in the low iron deposition category, especially in the absence of major iron deposition before its progression.

## Materials and methods

### Patients

Short-axis images of 40 patients were acquired using a clinical routine MGE cardiac MRI protocol between February 2009 and May 2016. The data were analyzed retrospectively, and the hospital review board waived the informed consent requirement. Motion correction was not performed, but images from ten patients were excluded due to respiratory motion blurring. The remainder of 30 included patients consisted of six patients with thalassemia (age average of 15 years with four male), six with hemochromatosis (age average of 55 years with two male), thirteen with suspected cardiomyopathy (age average of 46 years with eight male), and five patients with sickle cell disease (16 year, male), congenital dyserythropoietic anemia (25 year, male), chemotherapy (14 year), blood transfusion (19 year, male), or congenital heart disease (38 year, male).

### Cardiac iron assessment by magnetic resonance imaging

**Fig. 3:**
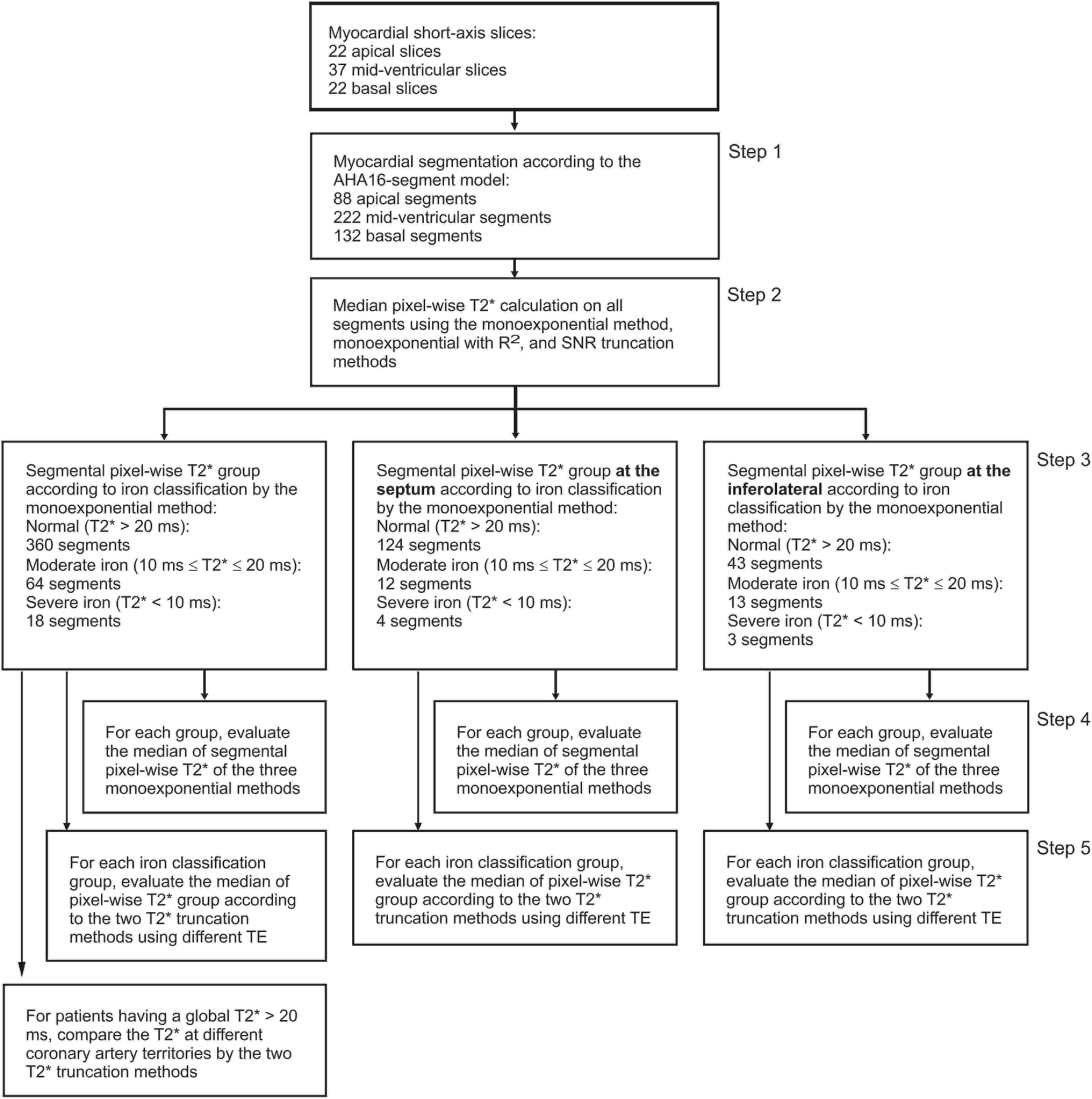
A flow chart showing data collection, selection, and calculation scheme for thirty patients. The number of segments and pixels as results are presented for clarity

A bright blood MGE sequence with a single breath-hold was used at 8 TEs with a repetition time of 200 ms and a flip angle of 20° on either one of two 1.5 T MRI systems (Siemens Medical Solutions, Erlangen, Germany) without enabling parallel imaging. From 2009 till 2011, the Avanto scanner (TEs of 2.59–18.20 at 2.23 ms increments, pixel bandwidth set at 814 Hz) was used on 18 patients. From 2012 till 2016, the Aera scanner (TEs of 2.69–18.86 at 2.31 ms increments, pixel bandwidth set at 815 Hz) was used on the subsequent 12 included patients. Depending on the field of view of 275–362 × 400 mm^2^ that reflects the patient size, a body matrix coil of 6–9 elements and a spine matrix coil of 12–24 elements were used. The MGE sequence used a number of excitation (NEX) of 1 and a reconstructed voxel size of 1.56 × 1.56 × 10 mm^3^. Phase resolution sampling of 50% was applied using 18–24 cardiac cycles per breath-hold with five segments in each heartbeat. Eighteen patients were scanned at the apical, mid-ventricular, and basal short-axis slices, while the rest were only scanned at the mid-ventricular slice. Four patients undergoing multiple follow-ups were acquired four complete short-axis slices at the three standard locations and three at mid-ventricular. Therefore, a total of 22 apical, 37 mid-ventricular, and 22 basal short-axis slices were acquired (Fig. 3, top).

Custom-written software (developed in MATLAB version 7.14, The MathWorks, Natick, MA, USA) was used to apply pixel-wise myocardial T2* quantification on all segments by a classic monoexponential fitting [27] as follows:

1 $$y = Ke^{{ - {\text{TE/T}}2^{*} }} ,$$

where *y*, *K*, TE, and T2* represent the signal intensity, a fitting constant, echo time of the MGE image series, and myocardium transverse relaxation time. A default non-linear least square curve fitting (MATLAB lsqcurvefit function) with trust-region-reflective was used to perform the fitting with a minimum *K*, and T2* constraint values were kept to be 0.

Two monoexponential truncation methods were adopted in this study. The SNR truncation method used pixel's SNR as a longer TE data point exclusion criterion with the SNR defined by the following equation [28]:

2 $$\mathrm{SNR}=\mathrm{NF}\frac{SI}{{\sigma }_{b}},$$

where NF, SI, σ_b_ represent the noise factor, pixel-wise signal intensity, and the standard deviation of a region of interest of air background, respectively. The noise factor accounts for underestimating noise derived from complex-magnitude data, assessed using the root of the sum of square (SoS) reconstruction and equals 0.71 for up to 32 coil elements according to Table 2 in the previous study [29].

Using the custom-written software, the SNR truncation method automatically excluded any pixel-wise TE data point with SNR below 2.5 [18] starts from longer TE data points of monoexponential analysis with maintaining TE below 10 ms or, in this study, four early TE points. Until TE 10 ms, the noise's influence is minimal and gives similar signal intensity for different NEX up to 32 (21) while maintaining enough information of the tissue's fast relaxation component [30, 31]. The R^2^ truncation used pixel's fitting *R*^2^ as the longer TE data point exclusion criterion. The exclusion will automatically exclude the point until the monoexponential fitting *R*^2^ exceeds a threshold value of 0.995 [10, 12] or, when not achieved, until four early TE points.

### Analysis and statistical methods

A cardiac radiologist with more than 10 years of experience performing semi-automatic LV epicardial and endocardial contours segmentation. The segmentation is conducted using custom-written software developed in MATLAB version 7.14; The MathWorks, Natick, MA, USA adapted from another study [32]. The AHA 16-segment model was used to segment the short-axis slices [27]. This model was used to analyze iron deposition in the myocardium as a whole (16 segments), at the suggested septum (i.e., apical septal, mid-ventricular anteroseptal, mid-ventricular inferoseptal, basal anteroseptal, and basal inferoseptal), and at inferolateral, that is prone to susceptibility artifact [13] (i.e., mid-ventricular inferolateral, and basal inferolateral).

Invasive iron deposition measurement was not conducted in this study. Therefore, iron deposition pathology was indicated by normal, moderate iron deposition, or severe iron deposition based on medians of pixel-wise T2* in the AHA segments. T2* classification for normal pathology (i.e., without iron deposition) was T2* > 20 ms, while for moderate iron deposition was 10 ms ≤ T2* ≤ 20 ms and for severe iron deposition was T2* < 10 ms [27, 33].

A flow chart of data assessment is shown in Fig. 3. The classic monoexponential fitting method (Eq. 1) was used to calculate the median pixel-wise T2* and its median absolute deviation (MAD) [27, 34] on each AHA segment (Fig. 3, step 2). The same T2* calculation was also conducted by the two alternative T2* monoexponential truncation methods. Then the segments were grouped according to the iron deposition classification (Fig. 3, step 3) at three different locations, i.e., global myocardium, septum, and inferolateral. Multiple median segmental pixel-wise T2* comparisons were made between the three methods for different iron deposition classification and different segment locations (Fig. 3, step 4). The pixel-wise T2* from the two truncation evaluation was grouped further to understand our observations on each iron deposition classification. Each group consists of pixel-wise T2* evaluated using different TE points from a minimum of 4 TE until 8 TE. (Fig. 3, step 5). Also, pixel-wise T2* analysis by the two truncation methods was conducted at different coronary artery territories, i.e., left anterior descending (LAD), right coronary artery (RCA), and left circumflex artery (LCx) territories [26] for patients without global iron deposition.

IBM SPSS Statistics software version 23 (IBM Corporation, Somer, NY, USA) was used for statistical testing. Multiple comparisons by one-way analysis of variance with Bonferroni post hoc test was used for normally distributed data. Kruskal–Wallis test analysis with Dun-Bonferroni post hoc test was used for non-normally distributed data. Shapiro–Wilk test was performed to test the normality of data distribution. *P* < 0.05 was considered statistically significant.

## Results

According to the classic monoexponential fitting method, from a total of 442 segments, 360 segments of 29 patients were classified as without iron deposition, 64 segments of 21 patients with moderate iron, and 18 segments of three patients with severe iron deposition, as shown in Fig. 3 and grouped in Table 1. From these segments, in the septal region, 124 segments of 29 patients were classified as without iron deposition, 12 segments of seven patients with moderate iron, and four segments of one patient with severe iron deposition. Meanwhile, in the inferolateral region, 43 segments of 25 patients were classified as without iron deposition, 13 segments of 10 patients with moderate iron, and three segments of two patients with severe iron deposition.

**Table 1:** Comparison of segmental pixel-wise T2* for three pixel-wise monoexponential fitting methods differentiated into categories of iron deposition

T2* quantification	ns	Monoexponential	*R*^2^ truncation	SNR truncation
		T2* (ms)	T2* (ms)	T2* (ms)
AHA 16 segments having				
T2* > 20 ms	360	31.96 ± 6.23	36.57 ± 6.95*	31.96 ± 6.22^‡^
10 ms ≤ T2* ≤ 20 ms	64	17.63 ± 2.15	22.47 ± 4.34*	17.74 ± 2.13^‡^
T2* < 10 ms	18	8.53 ± 1.46	8.22 ± 2.10	8.08 ± 2.02
Septal segments having				
T2* > 20 ms	124	36.52 ± 5.93	40.07 ± 5.89*	36.52 ± 5.93^‡^
10 ms ≤ T2* ≤ 20 ms	12	16.99 ± 2.65	22.34 ± 4.68	16.99 ± 2.65
T2* < 10 ms	4	6.97 ± 0.85	6.16 ± 0.26	6.21 ± 0.24
Inferolateral segments having				
T2* > 20 ms	43	25.73 ± 4.42	32.14 ± 5.78*	25.74 ± 4.41^‡^
10 ms ≤ T2* ≤ 20 ms	13	19.14 ± 1.21	26.14 ± 3.59*	19.52 ± 0.56
T2* < 10 ms	3	8.78 ± 0.38	9.54 ± 0.94	10.73 ± 0.52

Data are presented as median ± median absolute deviation. *ns* number of segments according to T2* classification by monoexponential fitting method, *SNR* signal-to-noise ratio, *AHA* American Heart Association. Both truncation methods were performed with a minimum of four echo times

*Dun-Bonferroni post hoc test *P* < 0.05 compared to the T2* classic monoexponential method

^‡^Dun-Bonferroni post hoc test *P* < 0.05 compared to the T2* monoexponential with R^2^ truncation method

Of 22 patients with data allowing for a complete AHA 16-segment model analysis, 20 had a global monoexponential T2* without iron deposition. One patient with congenital dyserythropoietic anemia had severe iron deposition in 14 segments, with only two segments classified as moderate. Another patient with thalassemia had a majority of moderate iron deposition segments with three severe iron deposition segments and only one segment at the borderline without iron classification. After a one-year follow-up, the patient had five segments classified as moderate and eleven segments without iron classification. Visual inspection showed a minor region of susceptibility artifact at the inferolateral region on 53 out of 59 collected segments.

**Fig. 4:**
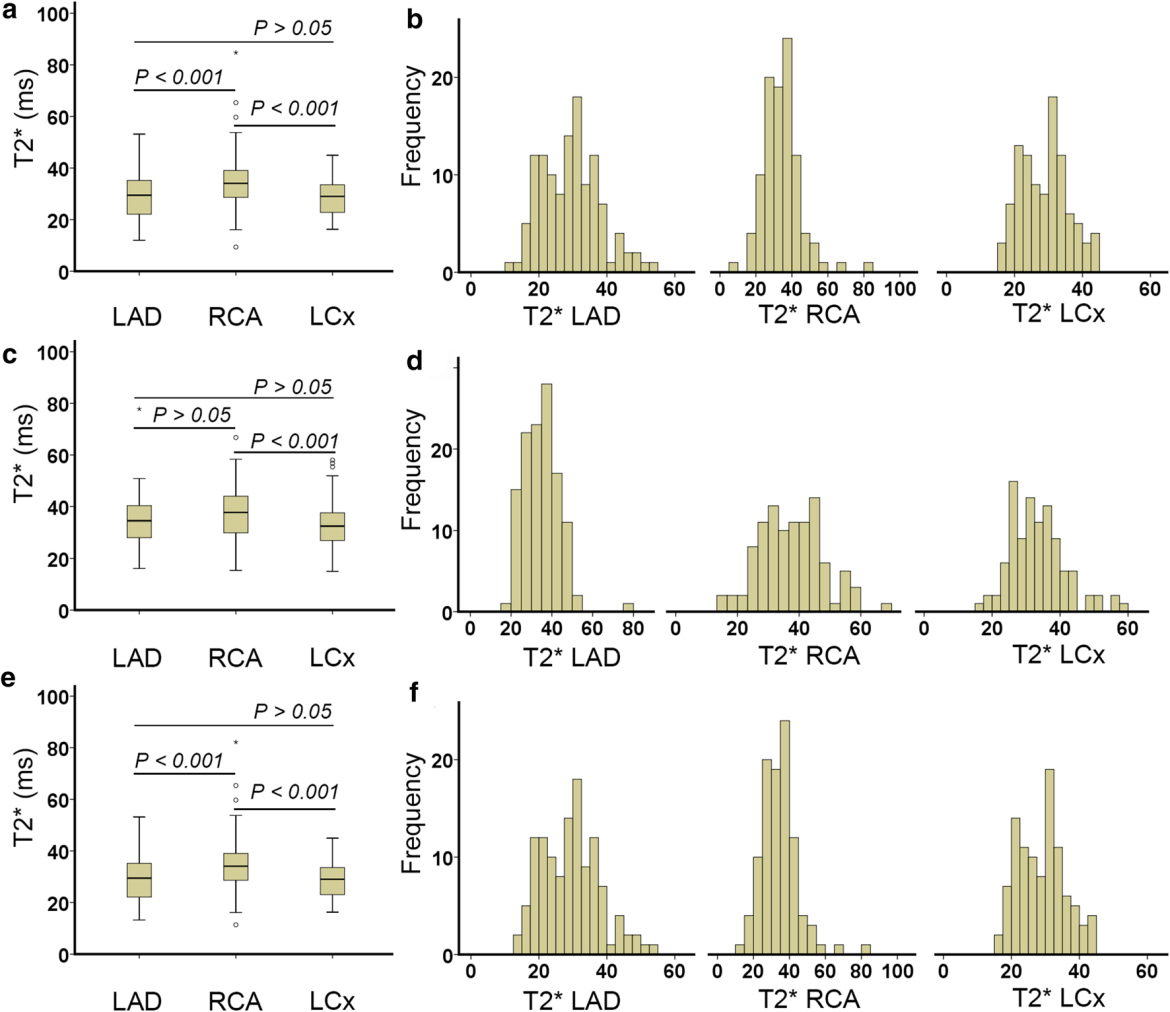
T2* heterogeneity of different coronary artery territories in patients without iron deposition evaluated by the classic monoexponential fitting (**a**) *R*^2^ truncation (**c**) and the SNR truncation (**e**) with its segmental T2* histogram (**b**, **d**, **f**), respectively

Evaluated by the three monoexponential fitting methods on 20 patients without global iron deposition, the RCA territory had a higher T2* value than the LAD and the LCx territories (*P* < 0.01), as shown in Fig. 4. However, when using *R*^2^ truncation, no significant difference was found between the RCA and the LAD (*P* > 0.05). No significant difference was found between the LCx and the LAD by the three fittings (*P* > 0.05).

**Fig. 5:**
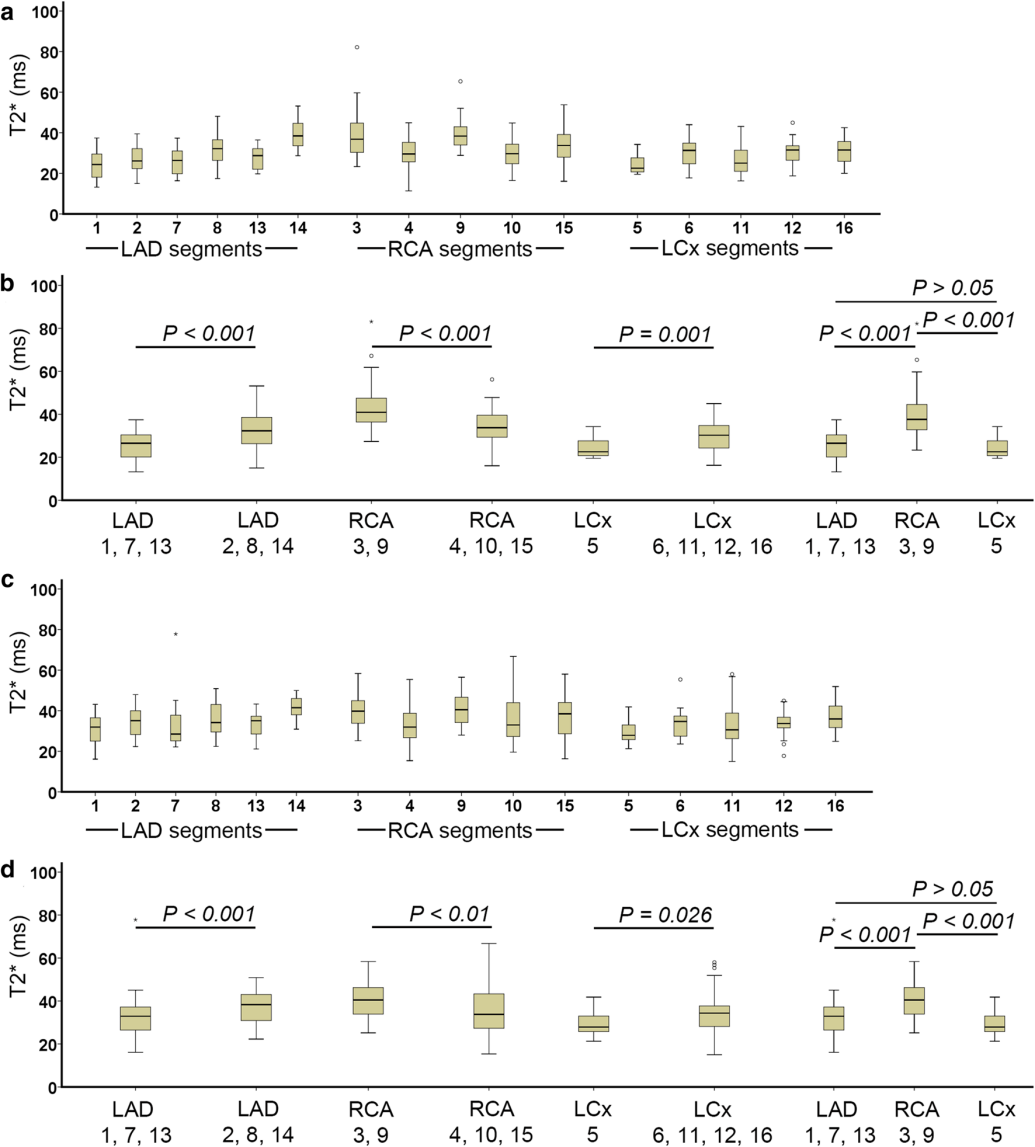
T2* heterogeneity in AHA 16-segment model in patients without iron deposition evaluated by the *R*^2^ truncation (**a**, **b**) and the SNR truncation (**c**, **d**). Territories comparison on b and d was conducted based on different coronary artery contributions to each segment according to Fig. 1b [26]

Histogram analysis on each territory in Fig. 4 showed multiple histogram peaks indicating multiple T2* components. Therefore, further analysis in Fig. 5a, c revealed T2* heterogeneity, using the two truncation methods, on AHA segments in each coronary artery territory. In Fig. 5b, d, similar T2* heterogeneity between the three coronary arteries was shown when comparing segments supported by a dominant coronary artery. A significant difference of T2* value was found on each territory between dominant coronary artery segments compared to segments supported by more arteries (*P* < 0.05).

**Fig. 6:**
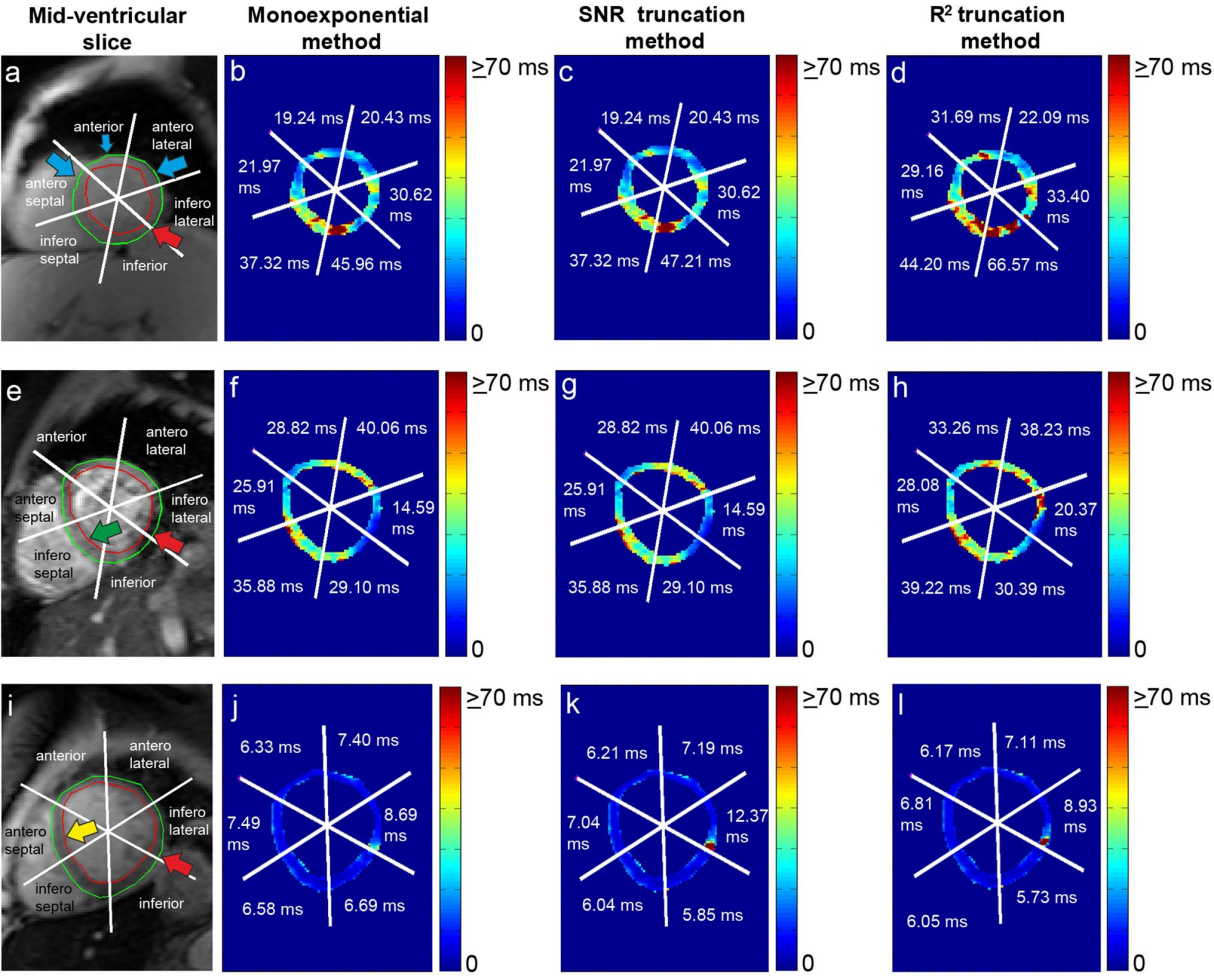
Pixel-wise T2* maps of the classic monoexponential method (**b**, **f**, **j**), the SNR truncation method (**c**, **g**, **k**), and the *R*^2^ truncation method (**d**, **h**, **l**) at mid-ventricular slices of Fig. 1 (**a**, **e**, **i**). Showing the indication of no iron deposition (green arrow head), susceptibility artifact at inferolateral (red arrow head), the progression of iron loading (blue arrow head), and severe iron deposition (yellow arrow head)

**Fig. 7:**
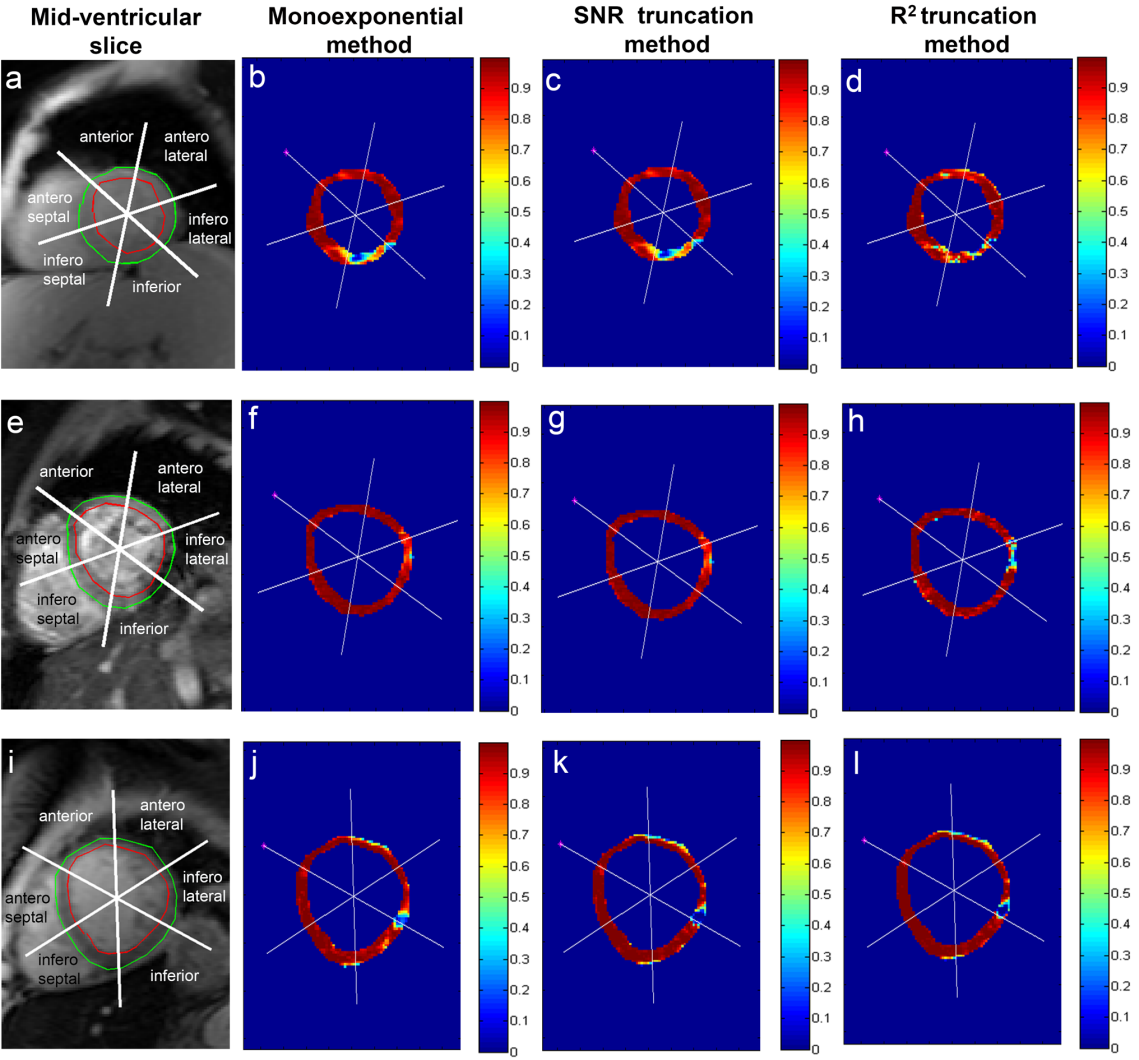
Pixel-wise *R*^2^ goodness of fitting maps of the classic monoexponential method (**b**, **f**, **j**), the SNR truncation method (**c**, **g**, **k**), and the *R*^2^ truncation method (**d**, **h**, **l**) at mid-ventricular slices of Figs. 1 and 6 (**a**, **e**, **i**)

Compared to the classic monoexponential and the SNR truncation method, the *R*^2^ truncation method consistently produced higher segmental T2* values in the absence of iron deposition at all investigated regions (*P* < 0.01), as shown in Table 1 and Fig. 6. Tables 2 and 3 express this significant difference between the two truncation methods on all AHA 16 segments and at recommended septal and at prone to artifact, inferolateral locations.The pixel-wise *R*^2^ goodness of fitting maps, presented in Fig. 7, show the two truncation methods' relatively high fitting performance on different myocardial regions. However, at the susceptibility artifact region, the *R*^2^ fitting of the two methods tends to reduce. On its majority pixels, the *R*^2^ truncation method used four early TE points to quantify T2*, i.e., 87.83%, 83.97%, and 93.99% of total pixels at the global myocardium, septal, and inferolateral regions, respectively. Meanwhile, the SNR truncation method used all TE points for the T2* quantification at the exact locations, i.e., 98.16%, 99.81%, and 92.81% of total pixels.

**Table 2:** Pixel-wise T2* quantification by the two monoexponential truncation methods evaluated by using different TE on all AHA 16 segments

		T2* (ms)				
		Using 8 TE	Using 7 TE	Using 6 TE	Using 5 TE	Using 4 TE
T2* > 20 ms^¥^	*R*^2^ truncation	32.99 ± 6.72 (5.42%)	31.96 ± 6.84 (1.84%)	32.89 ± 7.13 (2.13%)	33.30 ± 7.21 (2.78%)	37.71 ± 10.88 (87.83%)
(Total 100% pixels: 36,596)	SNR truncation	32.45 ± 8.37 (98.16%)	14.82 ± 2.82 (0.87%)	13.09 ± 2.81 (0.50%)	10.07 ± 2.35 (0.21%)	11.80 ± 4.95 (0.26%)
10 ms < T2* ≤ 20 ms^¥^	*R*^2^ truncation	14.60 ± 3.09 (6.03%)	16.72 ± 5.50 (1.80%)	20.14 ± 7.09 (2.42%)	21.80 ± 6.78 (3.20%)	23.24 ± 6.24 (86.56%)
(Total 100% pixels: 5787)	SNR truncation	18.28 ± 3.80 (88.92%)	12.55 ± 1.64 (4.56%)	12.92 ± 1.80 (2.78%)	10.84 ± 2.44 (2.07%)	7.82 ± 2.38 (1.66%)
T2* ≤ 10 ms^¥^	*R*^2^ truncation	7.67 ± 2.00 (9.25%)	6.47 ± 1.44 (2.89%)	7.03 ± 1.87 (3.78%)	6.13 ± 1.80 (5.04%)	8.40 ± 2.97 (79.03%)
(Total 100% pixels: 1903)	SNR truncation	14.45 ± 4.00 (19.92%)	11.39 ± 2.02 (7.41%)	11.67 ± 1.91 (7.83%)	8.89 ± 1.44 (11.25%)	5.97 ± 1.40 (53.60%)

Data are presented as median ± median absolute deviation. Data in parentheses are the percentage of quantified pixels from its total in each iron deposition classification

*TE* echo time, *SNR* signal to noise ratio

^¥^Pixels selection was based on T2* classification by the classic monoexponential fitting method on Table 1

**Table 3:** Pixel-wise T2* quantification by the two monoexponential truncation methods evaluated by using different TE at recommended septal and at prone to artifact, inferolateral locations

		T2* (ms)				
		Using 8 TE	Using 7 TE	Using 6 TE	Using 5 TE	Using 4 TE
Pixel-wise T2* at septal segments ^¥^						
T2* > 20 ms	*R*^2^ truncation	33.92 ± 6.67 (7.71%)	33.37 ± 7.49 (2.25%)	34.12 ± 6.95 (2.64%)	34.76 ± 7.45 (3.43%)	41.02 ± 11.15 (83.97%)
(Total 100% pixels: 14,027)	SNR truncation	35.73 ± 9.05 (99.81%)	14.13 ± 1.28 (0.15%)	11.53 ± 1.23 (0.02%)	13.06 ± 0.94 (0.01%)	47.91 (1 pixel)
10 ms < T2* ≤ 20 ms	*R*^2^ truncation	13.38 ± 2.81 (13.01%)	15.92 ± 4.23 (3.41%)	18.79 ± 7.04 (4.80%)	21.54 ± 5.92 (3.58%)	24.71 ± 5.35 (75.20%)
(Total 100% pixels: 1145)	SNR truncation	17.79 ± 3.74 (96.94%)	10.80 ± 1.65 (1.83%)	10.16 ± 0.41 (0.52%)	6.82 ± 0.30 (0.52%)	5.75 ± 0.14 (0.17%)
T2* ≤ 10 ms	*R*^2^ truncation	6.62 ± 1.20 (21.92%)	6.19 ± 1.66 (6.58%)	5.82 ± 1.54 (7.95%)	5.75 ± 0.95 (9.32%)	5.80 ± 1.18 (54.25%)
(Total 100% pixels: 365)	SNR truncation	14.34 ± 2.09 (16.16%)	11.09 ± 0.91 (4.66%)	8.12 ± 0.77 (3.56%)	7.23 ± 0.93 (11.23%)	5.29 ± 0.71 (64.38%)
Pixel-wise T2* at inferolateral segments ^¥^						
T2* > 20 ms	*R*^2^ truncation	28.57 ± 4.30 (1.74%)	28.52 ± 6.51 (1.10%)	23.51 ± 4.21 (1.49%)	30.19 ± 8.70 (1.69%)	32.80 ± 11.64 (93.99%)
(Total 100% pixels: 4090)	SNR truncation	27.83 ± 7.84 (92.81%)	14.22 ± 2.40 (2.96%)	13.21 ± 2.83 (2.54%)	9.02 ± 1.89 (0.93%)	10.42 ± 2.88 (0.76%)
10 ms < T2* ≤ 20 ms	*R*^2^ truncation	12.83 ± 4.36 (2.10%)	16.84 ± 5.75 (1.05%)	20.73 ± 7.11 (0.79%)	18.68 ± 6.24 (1.84%)	23.09 ± 7.92 (94.22%)
(Total 100% pixels: 1142)	SNR truncation	20.05 ± 4.93 (81.79%)	13.24 ± 2.02 (7.27%)	13.80 ± 2.10 (4.20%)	13.03 ± 2.67 (2.80%)	7.57 ± 2.42 (3.94%)
T2* ≤ 10 ms	*R*^2^ truncation	7.00 ± 0.44 (0.94%)	9.24 ± 1.33 (0.94%)	7.49 ± 0.01 (0.47%)	8.84 ± 1.36 (1.88%)	9.80 ± 3.01 (95.76%)
Total 100% pixels: 425)	SNR truncation	15.72 ± 5.21 (7.53%)	15.58 ± 3.49 (8.24%)	13.63 ± 2.41 (14.82%)	10.34 ± 2.03 (17.65%)	7.95 ± 1.97 (51.76%)

Data are presented as median ± median absolute deviation. Data in parentheses are the percentage of quantified pixels from its total in each iron deposition classification

*TE* echo time, *SNR* signal to noise ratio

^¥^Pixels selection was based on T2* classification by the classic monoexponential fitting method on Table 1

In moderate iron deposition classification at the global myocardium region, the T2* values from the *R*^2^ truncation method were significantly higher than the T2* resulted from the other two fitting methods (*P* < 0.001). Table 2 shows that the *R*^2^ truncation method used four TE points in the majority of 86.56% pixels to quantify T2* while the SNR truncation method used all TE points in 88.92% pixels. Meanwhile, at septal and inferolateral regions, even though a different use of TE points in the majority of pixels remains (Table 3), the T2* values produced by the two truncation methods are not significantly different (*P* > 0.05). Segmental T2* quantification by the three monoexponential fitting methods was not significantly different in severe iron deposition segments at any investigated region (*P* > 0.05). Table 2 shows that both truncation methods used four early TE values in a majority of pixels for quantifying T2*.

**Fig. 8:**
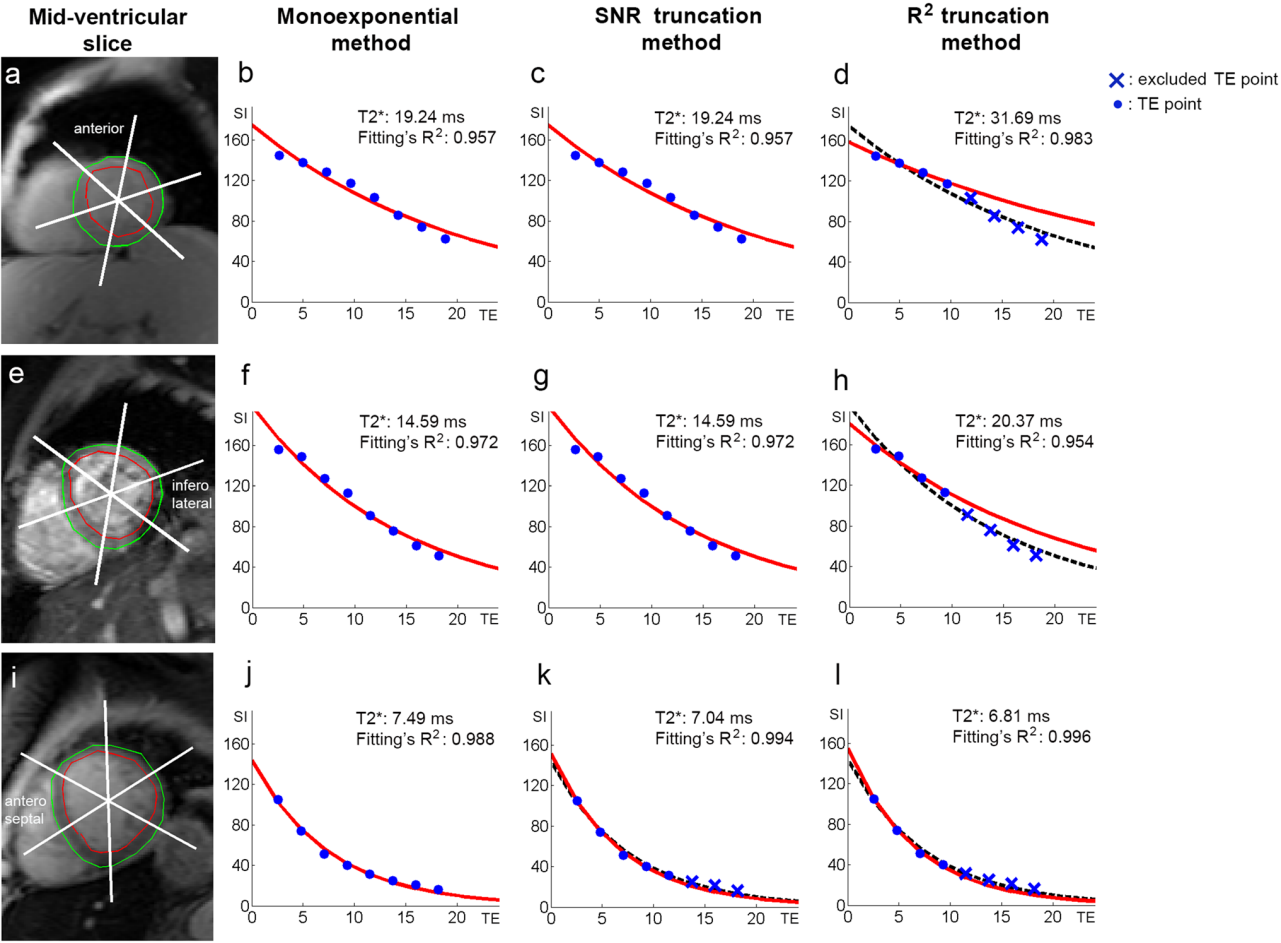
The comparison of segmental T2* monoexponential fitting by the classic monoexponential method (**b**, **f**, **j**), SNR truncation method (**c**, **g**, **k**), and *R*^2^ truncation method (**d**, **h**, **l**) at mid-ventricular anterior (**a**), inferolateral (**e**), and anteroseptal (**i**) of Fig. 1

In all iron deposition classification and at different analyzed locations, the elimination of later TE points increased the T2* values when quantified by the *R*^2^ truncation, as shown in Fig. 8, Tables 2, and 3. However, when the SNR truncation is used, the elimination of later TE points will decrease the quantified T2*.

## Discussion

This study demonstrated T2* heterogeneity in different circumferential regions according to coronary artery territories. T2* differences were also apparent when the analysis focused on the coronary arteries' contribution in supplying blood on segments. A consistent T2* heterogeneity by the two truncation methods was also found in the territories analysis based on its contribution on segments.

T2* variation occurred from different pixel-wise monoexponential curve fitting truncation methods. These apparent discrepancies apply either to the myocardium (AHA 16-segment model) or at the septal and inferolateral regions.. Therefore, the difference can influence clinical decision-making, especially in the myocardial region indicated with moderate iron deposition and without iron deposition, for the early treatment of patients with thalassemia, hemochromatosis, cardiomyopathy, and sickle cell disease.

It is known that some center prefers mid-ventricular septal to detect iron loading [2, 10, 14], while others choose to detect its progression at the global myocardium [1, 4, 8] with performing correction maps [35, 36]. Early detection of T2* is beneficial to understand its relation with iron distribution mechanisms on different diseases [37–42]. Meanwhile, both truncation methods produce similar results in identifying a severe iron loading region. Therefore, it is essential to understand the causes of the measurement discrepancies as a base for selecting the most appropriate analysis method to identify iron loading in a wide range of conditions.

The nature of R^2^ truncation likelihood in eliminating later TE points shows that the method is sensitive to pixel-wise signal intensity fluctuation to produce optimum R^2^ fitting. It is known that the source of the signal's fluctuation can be from partial volume voxel, cardiac motion, flow artifact, or fat and water phase oscillation [20]. Therefore, implementing the fitting's *R*^2^ until its maximum threshold, in this study 0.995, will push the TE point's elimination to meet the required *R*^2^ setting and leads to bias T2* measurement for iron deposition determination. An example is a possible missing chance of early iron deposition identification, as shown in the comparison between Fig. 6c, d at the anterior, anteroseptal, and anterolateral regions. A comparison between Fig. 8c, d, at the anterior region, show that the signal drops at early TE tend to be shallow at a higher T2* range [9, 33]. At the myocardial areas with higher T2*, this condition might be influenced by the in-phase and opposed phase fat signal that makes the TE signal fluctuate [43]. Therefore, excluding later TE reduces the constant *K* value of Eq. 1 and produces a higher T2* value. Some study has suggested avoiding TE points exclusion when classic monoexponential evaluation shows normal T2* range when performing *R*^2^ truncation [44].

TE point elimination's main idea was to eliminate the influence of signal plateau at longer TE due to noise and artifact [15, 22]. Therefore, the truncation method's implementation should reflect this idea by performing elimination only when the source of the signal plateau becomes apparent and not due to signal fluctuation. Moreover, eliminating TE points until their minimum early TEs does not always produce better T2* *R*^2^ fitting, as shown in the comparison between Fig. 8g, h. Analysis in Fig. 7g, h shows the reduction of the pixel-wise *R*^2^ fitting of the *R*^2^ truncation method in the presence of susceptibility artifact. TE points elimination can lead to a high T2* value, as shown in Fig. 6g, h at the inferolateral qwregion.

The SNR truncation eliminates later TE points when its signal's SNR is below a certain threshold. This procedure will dominantly eliminate TE points in the presence of signal plateau at longer TE. This truncation means that the method will selectively eliminate TE points when the signal's SNR is below the susceptibility artifact regions' threshold. Several studies have reported that TE truncation, at 1.5 T was mainly apparent after 10 ms [9, 10, 22, 31, 33, 45, 46]. Besides, aliasing effects due to rapid signal decay and limited TE data points also mainly occurred at TE after 10 ms [20]. Table 3 shows that in its application, in the susceptibility artifact region, i.e., inferolateral, the number of evaluated pixel-wise T2* by using fewer TE points is higher than the recommended artifact-free region, i.e., septal. This TE points’ selection showed the SNR truncation method's ability to detect the presence of possible artifacts.

At severe iron classification, even though truncated until 4 points, the similarity of T2* between the two truncated methods with the classic monoexponential is acquired due to the capture of fast relaxation component at early TE as shown in Fig. 8i–l. The bright-blood SNR truncation method has been reported to yield T2* values closest to those obtained by black-blood mode at severe iron identification [22]. It is known that the black-blood mode has the advantage of reducing the influence of artifacts and partial volume effect by suppressing the blood signal to produce clear endocardial and epicardial borders.

It is important to realize that T2* heterogeneity on circumferential regions can be explained due to the different contributions of coronary artery territories on each segment [26]. The previous study also mentioned that segments 1, 7, 13 were exclusively supported by LAD and are shown by the lower T2* threshold in Fig. 5 [26]. Further investigation in Fig. 5 showed that higher T2* at segments 2, 8, and 14 in the LAD territory is relevant to the RCA's higher contribution on the territory. At segments 2 and 8, higher T2* values might be influenced by segments 3 and 9, partly supported by the LAD and RCA, as shown in Fig. 2b. The T2* heterogeneity can be explained due to coronary collateral circulation, which provides alternative blood supply pathways in segment's supported by multiple coronary artery territories [47–49].

In the RCA territory, it seems that other territories also support segments 4 and 10. Therefore its T2* was lower compared to segments 3 and 9. Broader T2* range at segment 15 was explained due to the support of all territories at this segment. Meanwhile, in the LCx territory, segment 5 has lower T2* variability due to its exclusive support only by the LCx, as shown in Fig. 2b [26]. The rest LCx segments were supported by LAD and are explained by no significant difference between LAD and LCx territories in this study. It is known that the RCA has a longer artery length than the LAD and LCx, with a higher pressure gradient received from other arteries. Therefore, it receives higher collateral supplies than LAD and LCx, as shown with higher T2* in Figs. 4 and 5 [47].

Similar T2* heterogeneity by the two truncation methods on coronary artery territories and circumferential regions reflects a different iron deposition progression on different locations. This clear finding is observed when the analysis used the dominant coronary artery segments as suggested in Fig. 2b [26] rather than the traditional territories in Fig. 2a. The analysis of the coronary artery contribution on the circumferential regions might also be used to explain the heterogeneity of T1 value across circumferential regions observed by several studies [34, 50, 51]. Several post mortem and prospective studies [1, 11, 17] had reported a lower T2* value at the anterior, followed by inferior and lateral. Therefore, early detection of iron deposition is advised to start from these locations. The suggestion is also supported by the coronary artery analysis showing that LCx and LAD arteries had a lower T2* than RCA. It is known that LCx and LAD arteries were responsible for supplying blood to the most anterior and lateral segments while RCA supplies blood to inferior and septal segments [26]. Higher T2* value on RCA compared to other arteries indicates a lower progression of early iron deposition at the septal region, as detected previously [1, 4, 52].

When later TE points elimination is conducted to avoid any influence of signal plateau due to artifacts, the SNR truncation method is preferable to achieve the goal, as demonstrated in this study. However, it is essential to consider that the accuracy of the T2* values obtained with different fitting methods does not directly reflect tissue iron status [22]. A difference in T2* value, as a parameter to early identify iron deposition, by the two truncation methods is evident in this study. For that, comparing the two truncation methods with a different iron detection method [11, 17] in ex-vivo subjects would be advised at the higher end range of T2* values. Moreover, even though benefits from is lower variability on intraobserver and interobserver agreement, the validation of the two methods in higher magnetic field strengths is also needed due to its significantly higher susceptibility artifact at different locations [13, 53–55].

### Limitations

Here, the comparison of pixel-wise fitting methods and the influence of noise and artifact in T2* quantification of the myocardium was limited to the bright blood mode. Even though a low NEX was performed in this study, an attempt to increase NEX might reduce noise but at the expense of increasing scanning time [15, 44]. This study was conducted without enabling parallel imaging. Therefore the image SNR measurement can be straightforward on the MGE images. When parallel imaging is applied, a different approach in measuring the image's SNR is needed [18] and is beyond this study's scope.

Furthermore, rather than correcting for artifact contributions in the MGE data analysis, one might prefer to optimize the MGE imaging method to reduce and eliminate artifacts before the curve fitting (beyond the scope of this study). Another limitation is that this study used different scanners and slightly different TEs for acquiring the MGE series, relying on previous studies confirming the MRI sequence's reproducibility [2, 10]. The use of minimum TE close to 1 ms will increase the ability to capture the signal intensity fast relaxation component before the susceptibility artifact at mostly 10 ms [20, 46].

A limited number of patients each year who follow the MGE MRI clinical routine in our center also holds the strength of the analysis. The validation of the T2* measurement with the patient’s total body iron or other cardiac MR evaluations [56–58] is recommended to determine iron status and understand the progression of the disease. However, it is beyond the scope of this study.

## Conclusion

In conclusion, this study shows that focusing the coronary artery territories analysis based on the artery contribution on the segments produces a consistent T2* heterogeneity pattern by the two truncation methods. Moreover, the indication of severe and moderate iron deposition at the recommended septal region can be classified interchangeably by the two T2* truncation methods. Nevertheless, T2* differences remain in the region with no iron indication suggesting further investigation to avoid misleading early iron progression investigated by the two methods.
